# Psychosocial Outcomes in Patients Who Participated in a Hospital‐Based Family Involvement Program After Major Abdominal Oncological Surgery: A Preplanned Secondary Analysis of a Prospective Cohort Study

**DOI:** 10.1002/pon.70373

**Published:** 2026-01-01

**Authors:** Sani Kreca, Selma C. W. Musters, Susan van Dieren, Hanneke van der Wal‐Huisman, Johannes A. Romijn, Els J. M. Nieveen van Dijkum, Anne M. Eskes, the ARTIS Consortium

**Affiliations:** ^1^ Department of Surgery Amsterdam UMC Location University of Amsterdam Amsterdam the Netherlands; ^2^ Cancer Center Amsterdam, Treatment and Quality of Life Amsterdam the Netherlands; ^3^ Department of Surgery University of Groningen University Medical Center Groningen Groningen the Netherlands; ^4^ Department of Medicine Amsterdam UMC Location University of Amsterdam Amsterdam the Netherlands; ^5^ Faculty of Health Center of Expertise Urban Vitality Amsterdam University of Applied Sciences Amsterdam the Netherlands; ^6^ Menzies Health Institute Queensland and School of Nursing and Midwifery Griffith University Gold Coast Australia

**Keywords:** anxiety and depression, cancer, family caregiver, family centered care, family involvement, hospital admission, quality of life, satisfaction with care, sleep quality, surgery

## Abstract

**Background:**

Major abdominal cancer surgery can significantly affect patients' psychosocial wellbeing, including anxiety, depression, sleep quality, and self‐perceived health. Support from family caregivers during hospitalization may influence these outcomes.

**Aim:**

This study evaluates the psychosocial outcomes of a Family Involvement Program (FIP) for patients undergoing major abdominal cancer surgery.

**Methods:**

A secondary analysis was conducted using data from a patient‐preferred prospective cohort study. Patients who participated in the FIP alongside a family caregiver were compared to those who received usual care. The following psychosocial outcomes were assessed and analyzed using linear mixed‐effects models with stepwise backward selection: sleep quality, anxiety and depression (HADS), self‐perceived health (EQ‐VAS) and health related quality of life (EQ‐5D‐5L) index score. Satisfaction with care was analyzed using linear regression. Fidelity metrics included completion rates of fundamental and optional care activities and the number of caregivers staying overnight.

**Results:**

Patients in the FIP group reported significant higher sleep quality and significantly higher satisfaction with healthcare than those receiving usual care. No significant differences were found in anxiety, depression, or self‐perceived health between groups. Engagement in fundamental and optional care activities by family caregivers varied across activities and postoperative days, with 80%–90% of caregivers staying overnight.

**Conclusion:**

Family caregiver involvement during hospital admission for major abdominal cancer surgery improved sleep quality and satisfaction with care. Participation in the FIP did not significantly affect patients' anxiety, depression, or self‐perceived health. These findings suggest that family involvement programs may enhance the patients' psychosocial wellbeing during cancer recovery.

## 1 Background

The diagnosis of gastro‐intestinal cancer is a profoundly distressing event for patients, often triggering subclinical and clinical levels of anxiety and depression in up to 47.5% and 57% of cases, respectively [1, 2, 3]. These psychological burdens are particularly pronounced given the high mortality associated with gastrointestinal cancers [4, 5, 6]. These cancers are characterized by low survival rates and involve lengthy treatment trajectories and high‐risk surgical interventions, compounding the emotional and physical toll on patients [7, 8]. The resulting psychological distress not only diminishes patients' overall well‐being but is also associated with reduced quality of life [9] and impaired sleep quality [10]. Notably, poor sleep quality is reported in 60.7% of hospitalized cancer patients [11] and not only impacts emotional wellbeing [12, 13] but also affects cancer treatment outcomes [14] an survival [15, 16].

The psychological distress experienced by cancer patients not only impacts the patients themselves but also has consequences for healthcare systems. Elevated levels of anxiety and depression increase healthcare utilization, leading to more frequent hospital visits and prolonged stays [17, 18]. This increases the time and attention nurses must allocate to address not only the medical but also the psychosocial needs of patients, thereby straining resources further [19]. For example, high levels of distress are associated with an increased need for counseling and treatment, especially in the first year after being diagnosed with cancer [20, 21], which intensifies the workload for healthcare teams, particularly nurses, who are often the first point of contact for such support [22]. These demands, combined with a growing shortage of healthcare personnel—particularly nurses—further strain already overburdened resources [23]. Proactively addressing psychological symptoms in this patient group is crucial, not only to improve individual outcomes but also to reduce the systemic pressures these challenges impose on healthcare infrastructure [24].

One potential way to reduce psychological symptoms and illnesses as depression, anxiety, and stress is the involvement of family in healthcare [25, 26, 27]. Patients feel supported by the presence and involvement of family and need this during their cancer treatment [28]. Family involvement also improves the patients' reported satisfaction with care and sleep [29]. Although positive patient outcomes of family presence and involvement are increasingly described, family presence is not always a matter of course. Most hospitals still apply restricted visiting hours in adult healthcare, while literature shows that positive patient outcomes are more likely to be achieved as the involvement of family increases [30, 31, 32, 33].

Considering that family involvement can provide emotional support and enhance patient‐reported outcomes such as care satisfaction and sleep quality, it is crucial to examine its impact on patients' psychological well‐being, particularly through structured programs like the Family Involvement Program (FIP). This program was developed [34], tested [35, 36], and evaluated [37] in two university hospitals in the Netherlands. Evaluation of the FIP took place in our prospective cohort study, which assessed the FIP's effects on functional patient outcomes [38] and family caregiver outcomes [39]. Recently published articles reported that the primary outcome of the FIP, which was the 30‐day patient readmission rate, did not show a statistically significant reduction [38]. However, a secondary surgical outcome revealed a 16% reduction in the need for professional homecare after discharge, which was statistically significant [38]. Regarding family caregiver outcomes, caregivers' overall well‐being remained stable throughout the study period. Acceptable levels of caregiver burden were observed, and notably, 75% of family caregivers stated they would recommend the FIP to others [39]. In this analysis of the cohort study, we aim to evaluate the impact of the Family Involvement Program (FIP) on psychosocial outcomes such as sleep quality, anxiety, depression, self‐perceived health, and health‐related quality of life in patients receiving hospital care, compared to those receiving usual care. We hypothesized that patients who participate in FIP would experience higher satisfaction with care, lower levels of anxiety and depression, as well as higher sleep quality. These expectations were also described in advance in our logic model [34].

## 2 Method

### 2.1 Study Design

This is a preplanned secondary analysis of a multicenter prospective cohort study, named “Activating Relatives To get Involved in care after Surgery ‐1” (ARTIS‐1). The Medical Ethical Review Committee of Amsterdam UMC concluded that the Medical Research Involving Human Subjects Act does not apply to this study (reference number W19‐497 # 20.015). The study was conducted in accordance with the Declaration of Helsinki. All participants provided written informed consent prior to participation. The STrengthening the Reporting of OBservational studies in Epidemiology (STROBE) guideline was used as a reporting standard [40]. Initially, this study was designed as a randomized controlled trial (RCT) to minimize the risk of confounding and bias [41]. During the RCT period, we experienced substantial dropout due to strong preferences of patients and, in some cases, family caregivers. Reasons for dropout included that allocation to the control group was perceived as ethically unacceptable, as logistical arrangements had already been made to be present in the hospital during the FIP. To ensure feasibility and ethical acceptability, the study design was adapted to a prospective cohort design. This adaption is also well described in our published study protocol [41]. A comprehensive set of patient characteristics was collected to identify potential group imbalances.

### 2.2 Setting

This study was performed on surgical oncology wards of two university hospitals in Amsterdam and Groningen, the Netherlands. Patients were recruited from April 2019 until May 2022. Data were collected at several time points for each patient. Baseline data were collected one day prior to surgery. Additional data were collected during hospital admission, with follow‐up, scheduled at 30 and 90 days after surgery.

### 2.3 Participants

The detailed eligibility criteria for this study are described in the published study protocol [41] and the primary analysis of this study [38]. In brief, the study included adult patients scheduled for gastrointestinal cancer surgery, with an expected duration of postoperative hospital admission of at least 5 days. Participants needed to have sufficient proficiency in Dutch to understand and complete questionnaires. Patients could choose to join either the control group, which received standard postoperative care, or the intervention group, which involved participation in the Family Involvement Program (FIP) with one to three family caregivers of their choice. Caregivers had to be willing and able to stay in the hospital for a minimum of 8 hours per day during the first 5 days of the patient's admission.

### 2.4 Intervention

The FIP is a two‐component intervention consisting of education for healthcare personnel and training of the family caregiver by healthcare personnel to perform fundamental care activities such as breathing exercises, early mobilization, active orientation in time and place and oral care and intake. Optional care activities included helping with bathing, toilet assistance, wound dressing and administering anticoagulation injections. Also drain care, drain flushing, tube feeding and replacement of tape nose sticker were optional. Patients in the FIP had their family caregiver present during the hospital admission, with overnight stays being optional. The FIP is described in more detail in the study protocol [41] and the primary analysis of the cohort study [38]. The control group received usual postoperative care according to the enhanced recovery after surgery (ERAS) program. Control patients had the opportunity to receive visit throughout the day (11.00 a.m.–9.00 p.m.), according to the ward visiting policy.

### 2.5 Outcomes

The outcomes of this study were the effects of the FIP on patient sleep quality, overall satisfaction with care, anxiety and depression, self‐perceived health, and health related quality of life. Also fidelity metrics of the FIP were reported in order to interpretate the results. Patients' overall satisfaction with general care during hospital admission was measured using a visual analog scale (VAS), ranging from 0 to 10, with higher scores indicating greater satisfaction. This score was collected at hospital discharge.

A numeric rating scale (NRS), ranging from 0 to 10 was used daily during the first five days after surgery to obtain subjective scores on sleep quality. The NRS was selected for its simplicity in order to maximize response rates, given the large number of questionnaires administered to patients in this cohort study.

To measure anxiety and depression levels in patients, the validated Hospital Anxiety and Depression Scale (HADS) was used [42]. This scale consists of two subscales: anxiety and depression. Scores can range from 0 to 21. The score for the anxiety subscale is calculated as the sum of the points from the odd‐numbered items, while the score for the depression subscale is calculated as the sum of the points from the even‐numbered items. A high score on the HADS indicated more anxiety and depression during the last 4 weeks, without involving physical symptoms that are also distinctive for anxiety and depression [43].

Self‐perceived health was measured using the EuroQoL Visual Analog Scale (EQ‐VAS) [44]. Health‐related quality of life was measured with the EuroQol 5‐Dimension 5‐Level Health Questionnaire (EQ‐5D‐5L) questionnaire [45]. The EQ5D5L is presented by its dimensions, with figures illustrating changes for each time point during follow‐up, included in the supplementary material.

HADS, EQ‐VAS, and EQ‐5D‐5L questionnaires were collected one day prior surgery, on the day of discharge from the hospital, and 30 and 90 days after discharge. A research nurse ensured that the questionnaires were completed and collected during the FIP and throughout the follow‐up period.

Fidelity metrics for the FIP have been previously reported [39] and are summarized here to support interpretation of the clinical outcomes. Fidelity indicators captured the extent of family caregiver engagement in the intended components of the FIP, including completion rates of fundamental and optional care activities and the number of caregivers staying overnight during hospitalization. These indicators were recorded through family caregivers in diaries and cross‐checked by ward nurses and the research team (S.K., S.M.).

### 2.6 Other Measures

Health‐related outcomes, including sleep quality, quality of life, anxiety, depression, and satisfaction with care, may be influenced by sociodemographic patient characteristics. These factors include sex, age, educational attainment, marital status, parental status, employment (both paid and unpaid), and the availability of social support [46, 47, 48]. To adjust for these potential confounders, several patient, social, and clinical characteristics have been collected. These patient characteristics were collected through questionnaires at baseline. The validated Friendship scale was used as a measure of an individual's sense of social isolation and connectedness [49]. The Friendship scale ranges from 0 to 24, with higher scores indicating more social connectedness [49].

### 2.7 Statistical Analysis

Baseline patients clinical and social characteristics were presented as mean and standard deviation (SD) or as median and inter quartile range (IQR) for continuous variables, according to the distribution. Categorical and dichotomous variables were presented as frequencies and percentages. Baseline characteristics were tested for differences between the intervention and control group. For continuous variables with a normal distribution, the independent samples *t*‐test was used; for non‐normally distributed variables, the Mann‐Whitney *U* test was used. The Chi‐square test was used for categorical variables, and the Fisher exact test for dichotomous variables. Linear mixed effect models with stepwise backward selection were used to assess statistical differences in sleep quality, HADS, EQ‐VAS, and EQ‐5D‐5L between the groups. The EQ‐5D‐5L is reported with index scores. For satisfaction with care, linear regression analyses was used. Candidate baseline confounders (sex, marital status, and number of adult children ≥ 18 years) were specified a priori and entered into the linear mixed effect models and linear regression analyses. Covariates were evaluated using stepwise backward selection; non‐significant variables were removed. The Friendship Scale was excluded a priori to avoid multicollinearity with related social support constructs. Statistical analyses were conducted using R software (version 3.6.2; R Foundation for Statistical Computing). Missing data were not imputed in the analysis and were reported in the Results section. All analyses were performed on intention‐to‐treat basis. The study was powered on the primary outcome of the cohort study [41]. No formal sample size calculation was performed for the secondary outcomes presented in this analysis. Therefore, analyses of secondary outcomes should be interpreted as exploratory, and statistical power may have been limited.

## 3 Results

**FIGURE 1:**
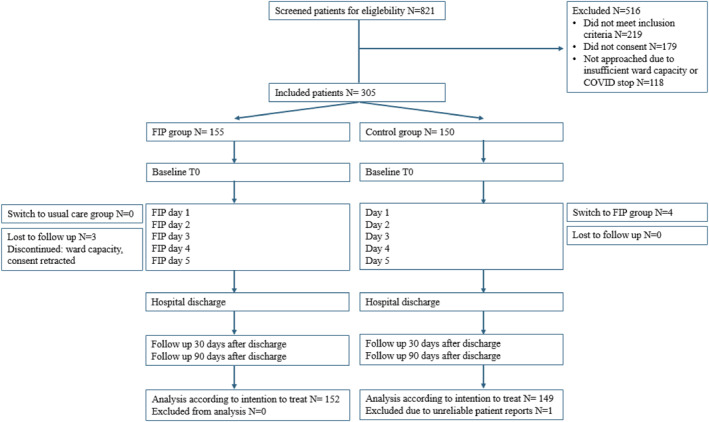
CONSORT study flow diagram, previously published in Musters et al. [38].

A total of 821 patients were scheduled for major abdominal cancer surgery with an expected admission of at least five days were eligible. Of these, 305 patients participated in this study, of with 155 patients in the FIP group and 150 patients in the control group. Figure 1 presents the study flow diagram.

### 3.1 Patient Characteristics

Table 1 presents the baseline characteristics of the patients. Significantly more male (*n* = 72,4%) than female (*n* = 27,6%) patients participated in the FIP group (*p* = 0.05). Furthermore, a significantly higher proportion of participants in de FIP group were married (*n* = 43%) compared to the control group (*n* = 34%; *p* = 0.01). There was also a significant difference in median number of adult children (≥ 18 years) between the groups, with a higher number of adult children in the FIP group (median = 2, IQR 1–2) compared to the control group (median = 2, IQR 0–2). Lastly, median scores on the Friendship Scale differed significantly between the two groups (*p* = 0.03). Other patient characteristics did not differ statistically between the FIP group and usual care group.

**TABLE 1:** Baseline characteristics patient.

	Family involvement program (*n* = 152)	Usual care (*n* = 149)	*p*‐value
Sex (*N*, %)			0.05^a^
Male	(110, 72.4)	(91, 61.1)	
Female	(42, 27.6)	(58, 38.9)	
Age (mean, SD)	(65.3, 9.9)	(64.7, 10.2)	0.59^b^
Highest level of formal education^e^ (*N*, %)			0.13^c^
Lower education	32 (21.1)	29 (19.5)	
Medium education	63 (41.4)	41 (27.5)	
Higher education	48 (31.6)	55 (36.9)	
Marital status (*N*, %)			0.01^c^
Married	125 (83.2)	99 (66.4)	
Not married^f^	24 (15.8)	43 (28.9)	
Children (*N*, %)			0.64^a^
Yes	(132, 85.5)	(124, 83.2)	
No	(22, 14.5)	(25, 16.8)	
Number of children < 18 years median, (IQR)	0, (0–0)	0, (0–0)	0.09^d^
Number of children > 18 years median, (IQR)	2, (1–2)	2, (0–2)	0.05^d^
Live‐in children (*N*, %)	5 (3.3)	11 (7.4)	0.76^a^
Yes	(27, 17.8)	(23, 15.4)	
No	(120, 78.9)	(115, 77.2)	
Number of live‐in children median, (IQR)	2, (1–2)	2, (1–2)	0.99^d^
Paid work (*N*, %)			0.21^c^
Yes, fulltime (36–40 h/week)	(37, 13.0)	(31, 10.9)	
Yes, part‐time	(17, 6.0)	(26, 9.1)	
No (including retirement)	(94, 33.0)	(80, 28.1)	
Paid work part‐time, hours/week median, (IQR)	25, (20–32)	24, (16–32)	0.91^d^
Unpaid work (*N*, %)	(10, 6.6)	(22, 14.8)	0.46^a^
Yes	(15, 9.9)	(18, 12.1)	
No	(127, 83.6)	(109, 73.2)	
Friendship scale median, (IQR)	1, (0–3)	2, (1–4)	0.03^d^

Abbreviations: EQ‐5D‐5L, euroqol 5‐dimension 5‐level; EQ VAS, EQ visual analog scale; HADS, hospital anxiety and depression scale; IQR, interquartile range; *N*, number; SD, standard deviation.

^a^ Fishers' exact test.

^b^ unpaired sample *t* test.

^c^ chi square test.

^d^ Mann‐Whitney *U* test.

^e^ Lower education level includes primary education plus the first 3 years of senior general secondary education (HAVO) and pre‐university secondary education (VWO); prevocational secondary education (VMBO) including lower secondary vocational training and assistant's training (MBO‐1). Medium education level includes upper secondary education (HAVO/VWO), basic vocational training (MBO‐2), vocational training (MBO‐3), and middle management and specialist education (MBO‐4). Higher education level includes associate degree programs, higher education (HBO/WO) Bachelor programs; 4‐year education at universities of applied sciences (HBO); Master degree programs at universities of applied sciences and at research universities (HBO, WO); and doctoral degree programs at research universities (WO). Missing data ranged between 2.0% and 26.8%.

^f^ Including: living common law, single, divorced, widowed.

### 3.2 Psychological Outcomes After Oncology Surgery

**FIGURE 2:**
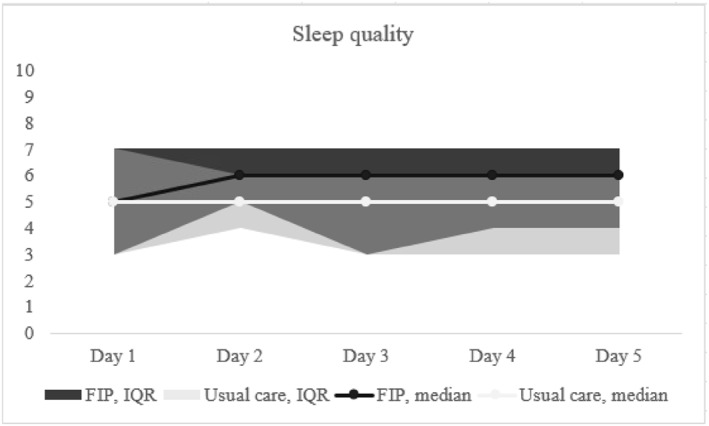
Sleep quality score of patients using a numeric rating scale from 0 to 10. *Missing values day 1 *N* = 97, day 2 *N* = 63, day 3 *N* = 69, day 4 *N* = 75, day 5 *N* = 92.

**FIGURE 3:**
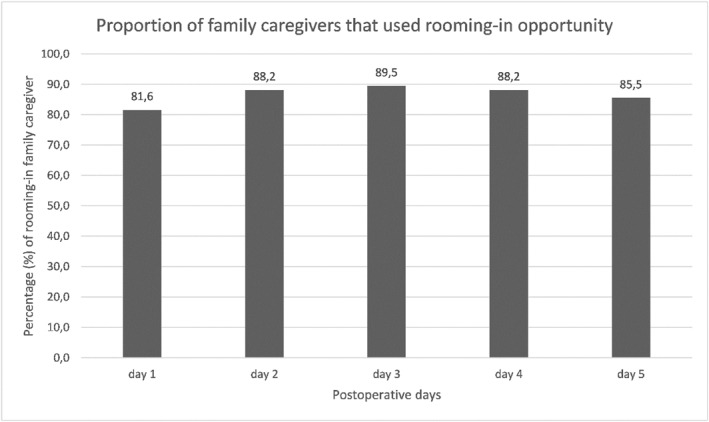
Proportion of family caregivers that used rooming‐in opportunity. Figure previously published [39].

The sleep score trends are shown in Figure 2. Patients in de FIP group had significantly higher sleep scores, with a median 6 (IQR 3–7) on the VAS, compared to the usual care group. Witch had a median 5 (IQR 3–7) on the VAS during the first 5 days after surgery (*p* = < 0.01). Family caregivers stayed overnight during the postoperative admission in 80%–90% of the patients in the FIP group (Figure 3), as previously reported [39].

Patient overall satisfaction with care was significantly higher in patients who participated in the FIP (median = 9; IQR 8–10) compared to patients who received usual care (median = 8; IQR 8–10) (*p* = 0.02). The HADS was divided into its two subscales, depression and anxiety. At baseline, higher anxiety levels were measured in the FIP group. During follow‐up, FIP patients did not score significant higher in depression or anxiety levels compared to the usual care group (Table 2). Self‐perceived health (Table 2) was not statistically different between the groups. Health‐related quality of life was also not statistically different between the groups (Table 2) and illustrated in more detail in the supplementary material (Supporting Information S1: Figure S1A/S1E). Outcome models were adjusted for baseline covariates identified as potential confounders (sex, marital status and number of adult children ≥ 18 years).

**TABLE 2:** Self‐perceived health and hospital anxiety and depression score outcomes.

	Baseline	At discharge	30 days after discharge	90 days after discharge	Estimate (95% CI)
HADS^a^ ‐ depression (median, IQR)					0.03 (0.61–0.67)
FIP	2 (1–4)	8 (5–10)	4 (2–6)	3 (2–6)	
Usual care	2 (0–4)	6 (5–7)	3 (1–6)	3 (1–8)	
HADS ‐ anxiety (median, IQR)					0.29 (0.3–0.91)
FIP	4 (1–6)	3 (1–6)	4 (1–5)	2 (1–4)	
Usual care	3 (1–5)	3 (1–5)	3 (1–5)	4 (1–7)	
EQ VAS (median, IQR)^b^					−1.30 (4.36–1.77)
FIP	80 (70–90)	65 (50–75)	70 (59–80)	75 (60–80)	
Usual care	80 (70–90)	65 (56–75)	70 (60–80)	80 (65–90)	
EQ5D5L index scores^c^					0.00 (−0.03–0.03)
FIP	0.885 (0.810–0.861)	0.775 (0.681–0.825)	0.791 (0.734–0.861)	0.869 (0.789–1.00)	
Usual care	0.887 (0.808–0.851)	0.783 (0.669–0.848)	0.848 (0.740–0.887)	0.870 (0.781–1.00)	

*Note:* Presented *p*‐values of outcomes are from adjusted linear mixed models. Outcome models were adjusted for baseline covariates retained by backward stepwise selection from the following candidates: sex, marital status, having children, and number of adult children.

Abbreviations: CI, Confidence Interval; EQ VAS, self‐perceived quality of life visual analog scale; HADS, hospital anxiety and depression scale.

^a^ Missing HADS: baseline FIP (*N* = 17) usual care (*N* = 28), discharge FIP (*N* = 26) usual care (*N* = 35), 30 days after discharge FIP (*N* = 50) usual care (*N* = 54), 90 days after discharge FIP (*N* = 136) usual care (*N* = 126).

^b^ Missing: baseline FIP (*N* = 15) usual care (*N* = 29), discharge FIP (*N* = 36) usual care (*N* = 42), 30 days after discharge FIP (*N* = 28) usual care (*N* = 32), 90 days after discharge FIP (*N* = 42) usual care (*N* = 35).

^c^ Missing scores were described in Supporting Information S1: Figures S1A–S1E.

## 4 Fidelity

**FIGURE 4:**
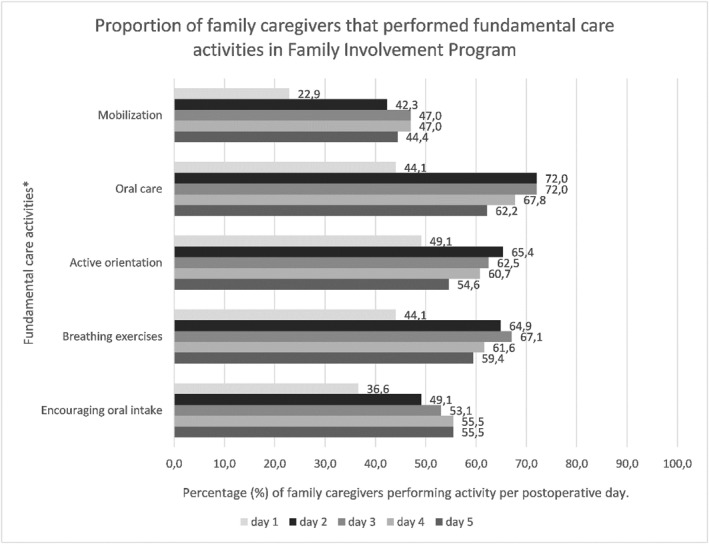
Proportion of family caregivers that performed fundamental care activities in the FIP. FIP, family involvement program. *Family caregivers needed to perform the fundamental care activities three times daily, with the exception of oral care, which was performed two times a day. The percentages presented in Figure 4 represent the average values across three (or two) distinct moments. Figure previously published [39].

**FIGURE 5:**
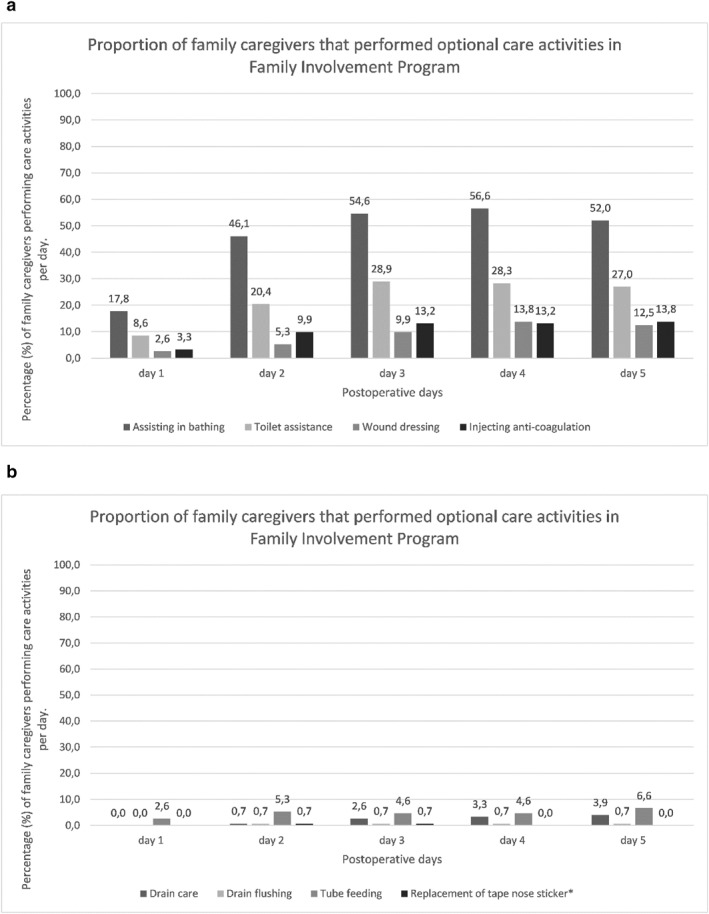
(a, b) Proportion of family caregivers that performed optional care activities in FIP. FIP, family involvement program. Figure previously published [39].

The previously published fidelity metrics [39] are presented in Figures 4, 5 and b. Figure 4 shows engagement with fundamental care activities over the postoperative days. Engagement with fundamental care activities by family caregivers increased over the first postoperative days, with the highest participation in oral care (62%–72%), active orientation (55%–65%) and breathing exercises (59%–67%) (Figure 4). Mobilization rose from 23% on day 1%–47% on day 4, while encouraging oral intake increased from 37% to approximately 56% (Figure 4). Optional care activities also showed increased engagement, with bathing assistance peaking at 56.6% on day 4, toilet assistance 28.9% on day 3, wound dressing at 13.8% on day 4 and administering anticoagulation injections at 13.8% on day 5 (Figures 5a–b).

## 5 Discussion

In this preplanned secondary analysis investigating the effects of the FIP, patients in the FIP group reported sufficient sleep quality, whereas patients in the usual care group reported insufficient sleep quality. Additionally, patients in the FIP group were more satisfied with care delivered compared to those who received usual care. For the remaining outcomes, there were no statistically significant differences between the groups in anxiety and depression scores, self‐perceived health, or health‐related quality of life. Fidelity metrics indicated that the level of participation varied across activities and postoperative days, with engagement in both fundamental and optional care activities generally increasing during the initial postoperative period; notably, most caregivers stayed overnight with the patient.

### 5.1 Proximity of Family Improves Sleep

The use of the single‐item Numerical Rating Scale (NRS) for assessing sleep quality in this study was a pragmatic decision, made to ensure feasibility and maximize response rates during the demanding early postoperative phase. Patients were asked to rate their sleep quality daily from postoperative day 1–6. At this stage of recovery, pain, fatigue, and analgesic medication can make completing longer, multidimensional questionnaires burdensome or unrealistic. Therefore, a brief, single‐item measure was considered most appropriate for this context. While the NRS is a simple, widely used patient‐reported outcome measure in various adult populations [50, 51], its psychometric properties in oncology patients have been sparsely evaluated in the literature. Oncology studies preferentially validate multi‐item measures such as the Insomnia Severity Index (ISI) [52] and the Pittsburgh Sleep Quality Index (PSQI) [53, 54, 55], as they provide a more in‐depth understanding of the multidimensional aspects of sleep quality, while NRS psychometrics in cancer largely concern pain [56], not sleep. As such, the interpretation of these sleep scores should focus on the patient's subjective perception and, where possible, be considered alongside additional qualitative assessments, as in our qualitative study addressing this cohort. There, patients described feeling safer in the hospital when their family caregiver was present, which may explain the subjectively higher sleep quality in patients who participated in the FIP. Although this difference in sleep scores seems small, improving from 5 in the control group to 6 in the FIP group, its clinical relevance is significant, as sleep scores in this study change from insufficient to sufficient sleep. Insufficient sleep quality is a well‐known and substantial problem in hospital patients [57, 58, 59], with up to 70% of hospitalized patients reporting insufficient sleep quality [60, 61, 62, 63]. The extend of this issue also carries an increased risk on several patient outcomes, such as delayed recovery after surgery [64, 65], increased risk of cardiovascular events [66], and cognitive function impairment [67, 68]. Therefore, improving sleep quality is relevant to enhance recovery after cancer surgery. However, some approaches, such as pharmacological interventions, are not without risk [69]. Serious adverse events, including cardiovascular events [69] and respiratory depression [70], have been reported when sleep disturbances are treated with pharmaceutics. Non‐pharmaceutical interventions can provide improved sleep quality and carry less risk on such serious adverse events [71]. Therefore, non‐pharmacological interventions to improve sleep, such as family involvement, could be a preferred method to increase sleep quality in clinical practice. The importance of proximity of family to improve quality of sleep in patients who underwent surgery is also described in recent literature on patients who underwent cardiac or cancer surgery [72, 73, 74] and can be explained by patients feeling safe and less stressed when their family is present during hospital admission, as described in our qualitative study [75].

### 5.2 Higher Satisfaction With Care

Patients who received usual care reported high satisfaction scores, assigning a score of 8 on the NRS, which ranges from 0 to 10. Notably, patients who participated in the (FIP) rated their overall satisfaction with care even higher. Similar scores were reported in a study by Schreuder et al. [36] and by Laitinen et al. [76]. Consistent with our findings, these studies demonstrated that satisfaction with care increased when family caregivers were involved in patient care. A possible explanation for higher satisfaction scores could be attributed to the family caregivers' role in providing emotional support [77]. Additionally, family caregivers may have facilitated better communication between healthcare professionals and patients by clarifying information [78]. Another possible explanation for the higher satisfaction in the FIP group is that patients may have perceived their care as more personalized and continuous [79]. However, the NRS rating scale for overall satisfaction with care does not specify the reason for satisfaction. Improving overall satisfaction with care in patients who already assign a high score presents a considerable challenge. The observed increase in overall satisfaction scores among patients in the FIP group suggests that the program provides substantial added value beyond standard care. This finding may reflect unmeasured but meaningful benefits of FIP, such as enhanced personalization of care.

### 5.3 Family Involvement in Care Does Not Lead to Increased Anxiety or Depression Levels

We found that patients who participated in the FIP had higher anxiety scores at baseline compared to the usual care group. Although statistical test were aimed at differences between the FIP and usual care group the development of the depression and anxiety scales over time is notable. Depression levels appear to be the higher at discharge in both groups. Nearing discharge can be a moment of stress for hospitalized patients [80]. These experiences of depression and anxiety during hospital admission often remain in the 6 months after discharge, emphasizing the relevance of psychological care during hospital admission [81]. Although high depression and anxiety levels often persist after discharge, it is notable that depression levels in the FIP group decrease to baseline level after discharge, and anxiety levels even decrease compared to baseline. Anxiety levels in the usual care group seem to remain stable or even show a slight increase after discharge. This might indicate that involving family in patientcare after discharge can alleviate depression and anxiety levels in patients.

When looking differences in HADS scores between the groups in the study, we found no higher levels of anxiety or depression in patients who participated in the FIP compared to the usual care group. This finding is relevant, as healthcare workers may perceive family involvement negatively due to concerns that it could disrupt patient rest, potentially leading to anxiety and depression [82, 83, 84, 85, 86]. FIP patients also did not have decreased anxiety and depression scores compared to the usual care group. This observation contrasts with existing literature, which documented decreased levels of anxiety and depression in cancer patients due to family involvement [25, 87].These contrasting findings should be interpreted while taking into account the observed intervention fidelity. Fidelity metrics showed that engagement in FIP components increased across postoperative days, which is consistent with patients' postoperative recovery and caregivers' progressive familiarization with care activities during hospitalization. As a result, exposure to the intervention was not uniform over time, and the reported outcomes represent average effects across varying levels of participation rather than effects attributable to a standardized or maximal implementation of the program. Comparable anxiety and depression scores between the FIP and the usual care group may be contributed to shared distress after being diagnosed with cancer, such as fears of death, uncertainty about survival, post‐surgery quality of life [88], and unmet needs [89]. These concerns may outweigh the potential benefits of family involvement on anxiety and depression as measured by the HADS.

Therefore, the HADS may not be sufficiently discriminatory to accurately quantify the psychological benefits of family involvement. Although family support does not entirely alleviate the fundamental worries linked to cancer and its treatment, which can impact HADS scores, it can play a vital role in regulating emotions and emotional processing [25, 90, 91, 92, 93]. The relevance of having family present was also described by patients in our qualitative study of the FIP [75], suggesting patients do experience psychological benefits from family involvement.

### 5.4 Clinical Implications

This study provides relevant clinical implications. First, improved patient satisfaction underscores the value of family involvement after major abdominal cancer surgery. Second, better sleep quality suggests a concrete psychosocial benefit that may aid recovery and emotional regulation. Third, while anxiety and depression levels did not differ significantly, caregiver presence may still play a meaningful role for some patients in coping with these symptoms, supporting broader psychosocial care goals.

In addition to psychosocial outcomes, the FIP was evaluated on functional surgical outcomes [33], family caregiver outcomes [34], patient safety [32], and the experiences of both family caregivers [30] and patients [46]. This comprehensive approach supports a well‐rounded understanding of the FIP's effects, contributing to its ongoing implementation in hospitals across the Netherlands.

As the Family Involvement Program is currently being implemented in hospitals across the Netherlands, these findings contribute to a growing foundation for sustainable, patient‐centered care strategies in psycho‐oncology.

### 5.5 Study Limitations

This study has limitations. Due to the non‐randomized cohort design, the possibility of selection bias cannot be fully excluded. Patients and families self‐selected into the FIP or usual care group, which could in theory have introduced systematic differences between groups. To indicate imbalanced differences between the groups, a comprehensive number of patient characteristics was collected. However, However, baseline characteristics showed some statistically significant differences between groups (sex, marital status, having children, and number of adult children); these variables were included as covariates in the linear mixed models to adjust for potential confounding, and therefore the risk of significant confounding is considered limited. Performance bias is expected to be limited, although it might have arisen in this study. Due to the design of the cohort study, healthcare professionals were aware if the patients participated in the FIP group or received usual care. This could have led to altered behavior, such as providing more emotional support to patients who did not have the emotional support of their family caregiver and received usual care. This could be an explanation of the similar HADS scores in both groups. Attrition bias seems unlikely since loss to follow‐up was minimal and mainly attributable to organizational reasons such as ward capacity. Overall, although the non‐randomized design may have introduced some degree of bias, the consistency of baseline characteristics and the very low attrition rate strengthen the validity of the findings.

A limitation in this study could be that the aimed secondary outcomes might be underpowered, as the sample size calculation was performed on primary outcomes of our cohort study [38]. A significant positive effect of the FIP on the patients' sleep was quantified. Therefore, this effect in improvement of sleep quality might even be more significant in clinical practice.

An explanation for higher anxiety scores at baseline in the FIP group might be found in sex. The FIP group had a higher proportion of male participants (72.4%) compared to the usual care group (61.1%). Previous research suggests that male patients tend to report higher anxiety levels than female patients [80], which could account for the elevated baseline anxiety observed in the FIP group. However, this does not explain the higher depression scores reported at hospital discharge in the FIP group, as female cancer patients generally exhibit slightly higher depression levels than male patients, as demonstrated in the systematic review by Vitale et al. [80].

Lastly, the extensive follow up period of patients during this cohort study is a strength. The follow‐up period of 90 days provides useful insights into how psychological outcomes in patients develop with family involvement during a long and stressful rehabilitation period, even after hospital admission [94].

## 6 Conclusions

Patients who participated in the family involvement program reported higher sleep quality and were more satisfied with the care received during hospital admission compared to patients who received usual care. The family involvement program did not lead to altered anxiety or depression levels. Self‐perceived health and health‐related quality of life were comparable between patients participating in the family involvement program and the usual care group. Fidelity metrics showed that caregiver engagement varied across activities and increased during the postoperative days. Based on these conclusions, hospitals could consider implementing family involvement programs to enhance patient experiences and satisfaction with care.

## Author Contributions

**Sani Kreca:** data curation, formal analysis, investigation, project administration, visualization, writing – original draft, writing – review and editing. **Selma C. W. Musters:** data curation, formal analysis, investigation, project administration, writing – review and editing. **Susan van Dieren:** methodology, validation, review and editing. **Hanneke van der Wal‐Huisman:** methodology, writing – review and editing. **Johannes A. Romijn:** methodology, writing – review and editing. **Els J. M. Nieveen van Dijkum:** conceptualization, methodology, supervision, writing – review and editing. **Anne M. Eskes:** conceptualization, methodology, supervision, writing – review and editing.

## Funding

The authors have nothing to report.

## Conflicts of Interest

The authors declare no conflicts of interest.

## Supporting information


Supporting Information S1

## Data Availability

The data that support the findings of this study are available from the corresponding author upon reasonable request.

## References

[1] V. Cheng , N. Oveisi , H. McTaggart‐Cowan , J. M. Loree , R. A. Murphy , and M. A. De Vera , “Colorectal Cancer and Onset of Anxiety and Depression: A Systematic Review and Meta‐Analysis,” Current Oncology 29, no. 11 (2022): 8751–8766, 10.3390/curroncol29110689. 36421342 PMC9689519

[2] W. Linden , A. Vodermaier , R. Mackenzie , and D. Greig , “Anxiety and Depression After Cancer Diagnosis: Prevalence Rates by Cancer Type, Gender, and Age,” Journal of Affective Disorders 141, no. 2–3 (2012): 343–351, 10.1016/j.jad.2012.03.025. 22727334

[3] Y. N. Peng , M. L. Huang , and C. H. Kao , “Prevalence of Depression and Anxiety in Colorectal Cancer Patients: A Literature Review,” International Journal of Environmental Research and Public Health 16, no. 3 (2019): 411, 10.3390/ijerph16030411. 30709020 PMC6388369

[4] Y. H. Wang , J. Q. Li , J. F. Shi , et al., “Depression and Anxiety in Relation to Cancer Incidence and Mortality: A Systematic Review and Meta‐Analysis of Cohort Studies,” Molecular Psychiatry 25, no. 7 (2020): 1487–1499, 10.1038/s41380-019-0595-x. 31745237

[5] M. Arnold , C. C. Abnet , R. E. Neale , et al., “Global Burden of 5 Major Types of Gastrointestinal Cancer,” Gastroenterology 159, no. 1 (2020): 335–349.e15, 10.1053/j.gastro.2020.02.068. 32247694 PMC8630546

[6] F. Bray , M. Laversanne , H. Sung , et al., “Global Cancer Statistics 2022: GLOBOCAN Estimates of Incidence and Mortality Worldwide for 36 Cancers in 185 Countries,” CA: A Cancer Journal for Clinicians 74, no. 3 (2024): 229–263, 10.3322/caac.21834. 38572751

[7] B. Housman , R. Flores , and D. S. Lee , “Narrative Review of Anxiety and Depression in Patients With Esophageal Cancer: Underappreciated and Undertreated,” Journal of Thoracic Disease 13, no. 5 (2021): 3160–3170, 10.21037/jtd-20-3529. 34164206 PMC8182527

[8] F. E. Van Beek , L. M. A. Wijnhoven , K. Holtmaat , et al., “Psychological Problems Among Cancer Patients in Relation to Healthcare and Societal Costs: A Systematic Review,” Psycho‐Oncology 30, no. 11 (2021): 1801–1835, 10.1002/pon.5753. 34228838 PMC9291760

[9] J. K. Hohls , H. H. Konig , E. Quirke , and A. Hajek , “Anxiety, Depression and Quality of Life‐A Systematic Review of Evidence From Longitudinal Observational Studies,” International Journal of Environmental Research and Public Health 18, no. 22 (2021): 12022, 10.3390/ijerph182212022. 34831779 PMC8621394

[10] Y. Jiang , T. Jiang , L. T. Xu , and L. Ding , “Relationship of Depression and Sleep Quality, Diseases and General Characteristics,” World Journal of Psychiatry 12, no. 5 (2022): 722–738, 10.5498/wjp.v12.i5.722. 35663298 PMC9150039

[11] M. Al Maqbali , M. Al Sinani , A. Alsayed , and A. M. Gleason , “Prevalence of Sleep Disturbance in Patients With Cancer: A Systematic Review and Meta‐Analysis,” Clinical Nursing Research 31, no. 6 (2022): 1107–1123, 10.1177/10547738221092146. 35484919 PMC9266067

[12] D. Tempesta , L. De Gennaro , V. Natale , and M. Ferrara , “Emotional Memory Processing is Influenced by Sleep Quality,” Sleep Medicine 16, no. 7 (2015): 862–870, 10.1016/j.sleep.2015.01.024. 26008959

[13] G. Jakobsen , K. H. Gjeilo , M. J. Hjermstad , and P. Klepstad , “An Update on Prevalence, Assessment, and Risk Factors for Sleep Disturbances in Patients With Advanced Cancer‐Implications for Health Care Providers and Clinical Research,” Cancers (Basel) 14, no. 16 (2022): 3933, 10.3390/cancers14163933. 36010925 PMC9406296

[14] M. M. Ghoneim and M. W. O'Hara , “Depression and Postoperative Complications: An Overview,” BMC Surgery 16, no. 1 (2016): 5, 10.1186/s12893-016-0120-y. 26830195 PMC4736276

[15] L. Strom , J. T. Danielsen , A. Amidi , A. L. Cardenas Egusquiza , L. M. Wu , and R. Zachariae , “Sleep During Oncological Treatment ‐ A Systematic Review and Meta‐Analysis of Associations With Treatment Response, Time to Progression and Survival,” Frontiers in Neuroscience 16 (2022): 817837, 10.3389/fnins.2022.817837. 35516799 PMC9063131

[16] K. P. Collins , D. A. Geller , M. Antoni , et al., “Sleep Duration is Associated With Survival in Advanced Cancer Patients,” Sleep Medicine 32 (2017): 208–212, 10.1016/j.sleep.2016.06.041. 28366336 PMC5428985

[17] J. E. Bosmans , M. C. de Bruijne , M. R. de Boer , H. van Hout , P. van Steenwijk , and M. W. van Tulder , “Health Care Costs of Depression in Primary Care Patients in the Netherlands,” Family Practice 27, no. 5 (2010): 542–548, 10.1093/fampra/cmq033. 20530527

[18] R. J. A. Linnemann , B. J. L. Kooijman , C. S. van der Hilst , et al., “The Costs of Complications and Unplanned Readmissions After Pancreatoduodenectomy for Pancreatic and Periampullary Tumors: Results From a Single Academic Center,” Cancers (Basel) 13, no. 24 (2021): 6271, 10.3390/cancers13246271. 34944890 PMC8699101

[19] L. Granek , O. Nakash , S. Ariad , S. Shapira , and M. Ben‐David , “Mental Health Distress: Oncology Nurses' Strategies and Barriers in Identifying Distress in Patients With Cancer,” Clinical Journal of Oncology Nursing 23, no. 1 (2019): 43–51, 10.1188/19.CJON.43-51. 30681995

[20] L. Tuominen , M. Stolt , R. Meretoja , and H. Leino‐Kilpi , “Effectiveness of Nursing Interventions Among Patients With Cancer: An Overview of Systematic Reviews,” Journal of Clinical Nursing 28, no. 13–14 (2019): 2401–2419, 10.1111/jocn.14762. 30585667

[21] A. Lewandowska , G. Rudzki , T. Lewandowski , and S. Rudzki , “The Problems and Needs of Patients Diagnosed With Cancer and Their Caregivers,” International Journal of Environmental Research and Public Health 18, no. 1 (2020): 87, 10.3390/ijerph18010087. 33374440 PMC7795845

[22] X. C. Lyu , H. J. Jiang , L. H. Lee , C. I. Yang , and X. Y. Sun , “Oncology Nurses' Experiences of Providing Emotional Support for Cancer Patients: A Qualitative Study,” BMC Nursing 23, no. 1 (2024): 58, 10.1186/s12912-024-01718-1. 38245735 PMC10800062

[23] E. E. T. Bolt , M. Ali , and J. Winterton , “Why Nurses Quit: Job Demands, Leadership and Voluntary Nurse Turnover in Adult Care in the Netherlands,” Social Science & Medicine 365 (2024): 117550, 10.1016/j.socscimed.2024.117550. 39637478

[24] B. M. Essue , N. Iragorri , N. Fitzgerald , and C. de Oliveira , “The Psychosocial Cost Burden of Cancer: A Systematic Literature Review,” Psycho‐Oncology 29, no. 11 (2020): 1746–1760, 10.1002/pon.5516. 32783287 PMC7754376

[25] S. Klankaew , S. Temthup , K. Nilmanat , and M. I. Fitch , “The Effect of a Nurse‐Led Family Involvement Program on Anxiety and Depression in Patients With Advanced‐Stage Hepatocellular Carcinoma,” Healthcare 11, no. 4 (2023): 460, 10.3390/healthcare11040460. 36832996 PMC9956382

[26] M. Koohi , M. Bagheri‐Nesami , R. Esmaeili , N. Mousavinasab , and H. Hosseini , “Effect of Family Participation in Primary Care Provision to Reduce Pain Anxiety Among Burn ICU Patients,” Journal of Mazandaran University of Medical Sciences 26, no. 146 (2017): 88–99, 10.1016/j.afjem.2020.11.003.

[27] P. Black , J. R. Boore , and K. Parahoo , “The Effect of Nurse‐Facilitated Family Participation in the Psychological Care of the Critically Ill Patient,” Journal of Advanced Nursing 67, no. 5 (2011): 1091–1101, 10.1111/j.1365-2648.2010.05558.x. 21214624

[28] K. R. Mitchell , K. J. Brassil , S. A. Rodriguez , et al., “Operationalizing patient‐centered Cancer Care: A Systematic Review and Synthesis of the Qualitative Literature on Cancer Patients' Needs, Values, and Preferences,” Psycho‐Oncology 29, no. 11 (2020): 1723–1733, 10.1002/pon.5500. 32715542 PMC7901502

[29] M. Fakhry and W. E. Mohammed , “Impact of Family Presence on Healthcare Outcomes and Patients' Wards Design,” Alexandria Engineering Journal 61, no. 12 (2022): 10713–10726: Elsevier, 10.1016/j.aej.2022.04.027.

[30] M. Park , T. T. Giap , M. Lee , H. Jeong , M. Jeong , and Y. Go , “Patient‐ and Family‐Centered Care Interventions for Improving the Quality of Health Care: A Review of Systematic Reviews,” International Journal of Nursing Studies 87 (2018): 69–83, 10.1016/j.ijnurstu.2018.07.006. 30056169

[31] K. Levoy , E. Rivera , M. McHugh , A. Hanlon , K. B. Hirschman , and M. D. Naylor , “Caregiver Engagement Enhances Outcomes Among Randomized Control Trials of Transitional Care Interventions: A Systematic Review and Meta‐Analysis,” Medical Care 60, no. 7 (2022): 519–529, 10.1097/mlr.0000000000001728. 35679175 PMC9202479

[32] M. Park and T. T. Giap , “Patient and Family Engagement as a Potential Approach for Improving Patient Safety: A Systematic Review,” Journal of Advanced Nursing 76, no. 1 (2020): 62–80, 10.1111/jan.14227. 31588602

[33] B. T. Bucher , M. Yang , R. Richards Steed , A. Fraser , S. R. G. Finlayson , and H. A. Hanson , “Geographic Proximity of Family Members and Healthcare Utilization After Complex Surgical Procedures,” Annals of Surgery 276, no. 4 (2022): 720–731, 10.1097/sla.0000000000005584. 35837896 PMC9463090

[34] A. M. Eskes , A. M. Schreuder , H. Vermeulen , E. J. M. Nieveen van Dijkum , and W. Chaboyer , “Developing an Evidence‐Based and Theory Informed Intervention to Involve Families in Patients Care After Surgery: A Quality Improvement Project,” International Journal of Nursing Science 6, no. 4 (2019): 352–361, 10.1016/j.ijnss.2019.09.006. PMC6838870 31728386

[35] A. M. Eskes , C. van Ingen , M. E. E. Horst , A. M. Schreuder , W. Chaboyer , and E. J. M. Nieveen van Dijkum , “The Experiences of Family Caregivers Who Participated in a Family Involvement Program After Cancer Surgery: A Qualitative Study,” European Journal of Oncology Nursing 49 (2020): 101835, 10.1016/j.ejon.2020.101835. 33120217

[36] A. M. Schreuder , A. M. Eskes , R. G. M. van Langen , S. van Dieren , and E. J. M. Nieveen van Dijkum , “Active Involvement of Family Members in Postoperative Care After Esophageal or Pancreatic Resection: A Feasibility Study,” Surgery 166, no. 5 (2019): 769–777, 10.1016/j.surg.2019.05.032. 31285045

[37] S. M. Kreca , I. S. Albers , S. C. W. Musters , et al., “The Effect of Family‐Centered Care on Unplanned Emergency Room Visits, Hospital Readmissions and Intensive Care Admissions After Surgery: A Root Cause Analysis From a Prospective Multicenter Study in the Netherlands,” Patient Safety in Surgery 18, no. 1 (2024): 14, 10.1186/s13037-024-00399-8. 38689336 PMC11061973

[38] S. C. W. Musters , S. M. Kreca , S. van Dieren , et al., “Surgical Outcomes in Surgical Oncology Patients Who Participated in a Family Involvement Program,” Surgery 176, no. 3 (2024): 826–834, 10.1016/j.surg.2024.05.004. 38897885

[39] S. C. W. Musters , S. M. Kreca , S. van Dieren , et al., “Family Caregiver Outcomes After Participating in a Hospital‐Based Family Involvement Program After Major Gastrointestinal Surgery: A Subgroup Analysis of a Patient Preferred Cohort Study,” International Journal of Surgery 110, no. 8 (2024): 4746–4753, 10.1097/js9.0000000000001473. 38626415 PMC11325895

[40] E. von Elm , D. G. Altman , M. Egger , et al., “The Strengthening the Reporting of Observational Studies in Epidemiology (STROBE) Statement: Guidelines for Reporting Observational Studies,” Lancet 370, no. 9596 (2007): 1453–1457, 10.1016/s0140-6736(07)61602-x. 18064739

[41] S. C. W. Musters , S. Kreca , S. van Dieren , et al., “Activating Relatives to Get Involved in Care After Surgery: Protocol for a Prospective Cohort Study,” JMIR Research Protocols 12 (2023): e38028, 10.2196/38028. 36440980 PMC9862329

[42] A. Vodermaier , W. Linden , and C. Siu , “Screening for Emotional Distress in Cancer Patients: A Systematic Review of Assessment Instruments,” Journal of the National Cancer Institute 101, no. 21 (2009): 1464–1488, 10.1093/jnci/djp336. 19826136 PMC3298956

[43] M. A. Annunziata , B. Muzzatti , E. Bidoli , et al., “Hospital Anxiety and Depression Scale (HADS) Accuracy in Cancer Patients,” Supportive Care in Cancer 28, no. 8 (2020): 3921–3926, 10.1007/s00520-019-05244-8. 31858249

[44] L. J. Cheng , R. L. Tan , and N. Luo , “Measurement Properties of the EQ VAS Around the Globe: A Systematic Review and Meta‐Regression Analysis,” Value in Health 24, no. 8 (2021): 1223–1233, 10.1016/j.jval.2021.02.003. 34372988

[45] Y. S. Feng , T. Kohlmann , M. F. Janssen , and I. Buchholz , “Psychometric Properties of the EQ‐5D‐5L: A Systematic Review of the Literature,” Quality of Life Research 30, no. 3 (2021): 647–673, 10.1007/s11136-020-02688-y. 33284428 PMC7952346

[46] O. Ribeiro , L. Teixeira , L. Araujo , C. Rodriguez‐Blazquez , A. Calderon‐Larranaga , and M. J. Forjaz , “Anxiety, Depression and Quality of Life in Older Adults: Trajectories of Influence Across Age,” International Journal of Environmental Research and Public Health 17, no. 23 (2020): 9039, 10.3390/ijerph17239039. 33291547 PMC7731150

[47] V. Lorant , D. Deliege , W. Eaton , A. Robert , P. Philippot , and M. Ansseau , “Socioeconomic Inequalities in Depression: A Meta‐Analysis,” American Journal of Epidemiology 157, no. 2 (2003): 98–112, 10.1093/aje/kwf182. 12522017

[48] J. K. Djernes , “Prevalence and Predictors of Depression in Populations of Elderly: A Review,” Acta Psychiatrica Scandinavica 113, no. 5 (2006): 372–387, 10.1111/j.1600-0447.2006.00770.x. 16603029

[49] G. Hawthorne , “Measuring Social Isolation in Older Adults: Development and Initial Validation of the Friendship Scale,” Social Indicators Research 77, no. 3 (2005): 521–548, 10.1007/s11205-005-7746-y.

[50] S. Stander , F. Fofana , C. Dias‐Barbosa , et al., “The Sleep Disturbance Numerical Rating Scale: Content Validity, Psychometric Validation, and Meaningful Within‐Patient Change in Prurigo Nodularis,” Dermatologic Therapy 13, no. 7 (2023): 1587–1602, 10.1007/s13555-023-00962-8. PMC10307761 37329468

[51] J. Puelles , F. Fofana , D. Rodriguez , et al., “Psychometric Validation and Responder Definition of the Sleep Disturbance Numerical Rating Scale in Moderate‐to‐Severe Atopic Dermatitis,” British Journal of Dermatology 186, no. 2 (2022): 285–294, 10.1111/bjd.20783. 34608623 PMC9299666

[52] M. H. Savard , J. Savard , S. Simard , and H. Ivers , “Empirical Validation of the Insomnia Severity Index in Cancer Patients,” Psycho‐Oncology 14, no. 6 (2005): 429–441, 10.1002/pon.860. 15376284

[53] M. Sohrabi , A. Gholami , P. Hassanzadeh , et al., “Examining the Factor Structure of the Pittsburgh Sleep Quality Index and Its Determinants Among GI Cancer Patients,” BMC Cancer 24, no. 1 (2024): 1577, 10.1186/s12885-024-13347-7. 39725909 PMC11670428

[54] A. Buttner‐Teleaga , Y. T. Kim , T. Osel , and K. Richter , “Sleep Disorders in Cancer‐A Systematic Review,” International Journal of Environmental Research and Public Health 18, no. 21 (2021): 11696, 10.3390/ijerph182111696. 34770209 PMC8583058

[55] M. Friedrich , T. Schulte , M. Malburg , and A. Hinz , “Sleep Quality in Cancer Patients: A Common Metric for Several Instruments Measuring Sleep Quality,” Quality of Life Research 33, no. 11 (2024): 3081–3091, 10.1007/s11136-024-03752-7. 39102095 PMC11541315

[56] W. H. Oldenmenger , P. J. de Raaf , C. de Klerk , and C. C. van der Rijt , “Cut Points on 0‐10 Numeric Rating Scales for Symptoms Included in the Edmonton Symptom Assessment Scale in Cancer Patients: A Systematic Review,” Journal of Pain and Symptom Management 45, no. 6 (2013): 1083–1093, 10.1016/j.jpainsymman.2012.06.007. 23017617

[57] S. Seid Tegegne and E. Fenta Alemnew , “Postoperative Poor Sleep Quality and Its Associated Factors Among Adult Patients: A Multicenter Cross‐Sectional Study,” Annals of Medicine and Surgery 74 (2022): 103273, 10.1016/j.amsu.2022.103273. 35145662 PMC8819123

[58] E. S. van den Ende , P. Burger , M. Keesenberg , H. Merten , R. Gemke , and P. W. B. Nanayakkara , “Patient‐Nurse Agreement on Inpatient Sleep and Sleep Disturbing Factors,” Sleep Medicine X. 4 (2022): 100047, 10.1016/j.sleepx.2022.100047. 35572156 PMC9097718

[59] P. Burger , E. S. Van den Ende , W. Lukman , et al., “Sleep in Hospitalized Pediatric and Adult Patients ‐ A Systematic Review and Meta‐Analysis,” Sleep Medicine X 4 (2022): 100059, 10.1016/j.sleepx.2022.100059. 36406659 PMC9672415

[60] H. M. Wesselius , E. S. van den Ende , J. Alsma , et al., “Quality and Quantity of Sleep and Factors Associated With Sleep Disturbance in Hospitalized Patients,” JAMA Internal Medicine 178, no. 9 (2018): 1201–1208, 10.1001/jamainternmed.2018.2669. 30014139 PMC6142965

[61] S. N. L. Y. Binte Arman , V. Lopez , and S. H. Lim , “Subjective Sleep Quality Among Hospitalised Adult Patients: An Observational, Cross‐Sectional Study,” in Proceedings of Singapore Healthcare, (2022).

[62] C. Adams , R. Harrison , A. Schembri , M. Junge , and R. Walpola , “The Silent Threat: Investigating Sleep Disturbances in Hospitalized Patients,” International Journal for Quality in Health Care 36, no. 2 (2024): mzae042, 10.1093/intqhc/mzae042. 38727537 PMC11107945

[63] S. Kulpatcharapong , P. Chewcharat , K. Ruxrungtham , et al., “Sleep Quality of Hospitalized Patients, Contributing Factors, and Prevalence of Associated Disorders,” Sleep Disorders 2020 (2020): 8518396–8518397, 10.1155/2020/8518396. 32308998 PMC7157800

[64] S. Rampes , K. Ma , Y. A. Divecha , A. Alam , and D. Ma , “Postoperative Sleep Disorders and Their Potential Impacts on Surgical Outcomes,” Journal of Biomedical Research 34, no. 4 (2019): 271–280, 10.7555/jbr.33.20190054. 32519977 PMC7386412

[65] R. M. Sipila and E. A. Kalso , “Sleep Well and Recover Faster With less Pain‐A Narrative Review on Sleep in the Perioperative Period,” Journal of Clinical Medicine 10, no. 9 (2021): 2000, 10.3390/jcm10092000. 34066965 PMC8124518

[66] V. K. Chattu , M. D. Manzar , S. Kumary , D. Burman , D. W. Spence , and S. R. Pandi‐Perumal , “The Global Problem of Insufficient Sleep and Its Serious Public Health Implications,” Healthcare 7, no. 1 (2018): 1, 10.3390/healthcare7010001. 30577441 PMC6473877

[67] A. Takamino , M. Kotoda , Y. Nakadate , S. Hishiyama , T. Iijima , and T. Matsukawa , “Short Sleep Duration on the Night Before Surgery is Associated With Postoperative Cognitive Decline in Elderly Patients: A Prospective Cohort Study,” Frontiers in Aging Neuroscience 13 (2021): 821425, 10.3389/fnagi.2021.821425. 35153727 PMC8831239

[68] J. Kang , Y. S. Cho , M. Lee , et al., “Effects of Nonpharmacological Interventions on Sleep Improvement and Delirium Prevention in Critically Ill Patients: A Systematic Review and Meta‐Analysis,” Australian Critical Care 36, no. 4 (2023): 640–649, 10.1016/j.aucc.2022.04.006. 35718628

[69] F. De Crescenzo , G. L. D'Alo , E. G. Ostinelli , et al., “Comparative Effects of Pharmacological Interventions for the Acute and Long‐Term Management of Insomnia Disorder in Adults: A Systematic Review and Network Meta‐Analysis,” Lancet 400, no. 10347 (2022): 170–184, 10.1016/S0140-6736(22)00878-9. 35843245

[70] S. Kanji , A. Mera , B. Hutton , et al., “Pharmacological Interventions to Improve Sleep in Hospitalised Adults: A Systematic Review,” BMJ Open 6, no. 7 (2016): e012108, 10.1136/bmjopen-2016-012108. PMC4986185 27473952

[71] A. D. Beswick , V. Wylde , W. Bertram , and K. Whale , “The Effectiveness of Non‐Pharmacological Sleep Interventions for Improving Inpatient Sleep in Hospital: A Systematic Review and Meta‐Analysis,” Sleep Medicine 107 (2023): 243–267, 10.1016/j.sleep.2023.05.004. 37257367

[72] S. E. Zahra Abbasi , R. Javanbakhtian , and M. Sedehi , “Effect of Family Centered Care on Sleep Quality of Post‐CABG Patients,” Journal of Biochemical Technology (2018): 55–61.

[73] X. Li , L. Chen , B. Lei , and C. Xie , “Home‐Based Psychological Nursing Interventions for Improvement of Sleep Quality and Psychological Health in Patients With Hypopharyngeal Carcinoma Undergoing Surgical Resections: A Randomized Trial,” Annals of Palliative Medicine 10, no. 12 (2021): 12347–12357, 10.21037/apm-21-3029. 35016426

[74] H. Bagheri , F. Norouzi , M. Maleki , et al., “The Effect of Increasing Duration of Family Members' Presence on Sleep Status in Patients With Acute Coronary Syndrome in Cardiac Care Unit: A Randomized Controlled Trial,” Nursing Open 11, no. 3 (2024): e2114, 10.1002/nop2.2114. 38424637 PMC10904766

[75] S. M. Kreca , S. C. W. Musters , M. E. E. Horst , C. van Ingen , E. J. M. Nieveen van Dijkum , and A. M. Eskes , “The Experiences of Patients Who Participated in a Family Involvement Program After Abdominal Cancer Surgery: An Interpretative Phenomenological Analysis,” Gastroenterology Nursing (2024). 10.1097/SGA.0000000000000857 39575933

[76] P. Laitinen , P. Merilainen , and S. Sinkkonen , “Quality of Elderly‐Patient Care: An Interrupted Time Series Study,” International Journal of Nursing Practice 2, no. 3 (1996): 129–137, 10.1111/j.1440-172x.1996.tb00038.x. 9265606

[77] S. M. Kreca , S. C. W. Musters , M. E. E. Horst , C. van Ingen , E. J. M. Nieveen van Dijkum , and A. M. Eskes , “The Experiences of Patients Who Participated in a Family Involvement Program After Abdominal Cancer Surgery: An Interpretative Phenomenological Analysis Society of Gastroenterology Nurses and Associates,” (March‐April 2023). 10.1097/SGA.0000000000000857 39575933

[78] M. den Herder‐van der Eerden , J. Hasselaar , S. Payne , et al., “How Continuity of Care is Experienced Within the Context of Integrated Palliative Care: A Qualitative Study With Patients and Family Caregivers in Five European Countries,” Palliative Medicine 31, no. 10 (2017): 946–955, 10.1177/0269216317697898. 28659022

[79] W. H. Organization , “Continuity and Coordination of Care,” in A Practice Brief to Support Implementation of the WHO Framework on Integrated people‐centred Health Services (Geneva, 2018).

[80] F. Bt Walker , D. H. Novack , D. L. Kaiser , A. Knight , and P. Oblinger , “Anxiety and Depression Among Medical and Surgical Patients Nearing Hospital Discharge,” Journal of General Internal Medicine 2, no. 2 (1987): 99–101, 10.1007/bf02596305. 3559782

[81] H. Sveinsdottir , S. Zoega , B. Ingadottir , and K. Blondal , “Symptoms of Anxiety and Depression in Surgical Patients at the Hospital, 6 Weeks and 6 Months Postsurgery: A Questionnaire Study,” Nursing Open 8, no. 1 (2021): 210–223, 10.1002/nop2.620. 33318829 PMC7729539

[82] D. Berti , P. Ferdinande , and P. Moons , “Beliefs and Attitudes of Intensive Care Nurses Toward Visits and Open Visiting Policy,” Intensive Care Medicine 33, no. 6 (2007): 1060–1065, 10.1007/s00134-007-0599-x. 17384930

[83] D. M. Berwick and M. Kotagal , “Restricted Visiting Hours in ICUs: Time to Change,” JAMA 292, no. 6 (2004): 736–737, 10.1001/jama.292.6.736. 15304472

[84] H. Hurst , J. Griffiths , C. Hunt , and E. Martinez , “A Realist Evaluation of the Implementation of Open Visiting in an Acute Care Setting for Older People,” BMC Health Services Research 19, no. 1 (2019): 867, 10.1186/s12913-019-4653-5. 31752862 PMC6873458

[85] B. Hetland , N. McAndrew , J. Perazzo , and R. Hickman , “A Qualitative Study of Factors That Influence Active Family Involvement With Patient Care in the ICU: Survey of Critical Care Nurses,” Intensive and Critical Care Nursing 44 (2018): 67–75, 10.1016/j.iccn.2017.08.008. 29169879 PMC5736422

[86] E. M. Melo , P. L. Ferreira , R. A. Lima , and D. F. Mello , “The Involvement of Parents in the Healthcare Provided to Hospitalzed Children,” Revista Latino‐Americana de Enfermagem 22, no. 3 (2014): 432–439, 10.1590/0104-1169.3308.2434. 25029054 PMC4292621

[87] J. Duong , G. Wang , G. Lean , D. Slobod , and M. Goldfarb , “Family‐Centered Interventions and Patient Outcomes in the Adult Intensive Care Unit: A Systematic Review of Randomized Controlled Trials,” Journal of Critical Care 83 (2024): 154829, 10.1016/j.jcrc.2024.154829. 38759579

[88] Bethesda . Supportive and Palliative Care Editorial Board, PDQ Adjustment to Cancer (National Cancer Institute, 2024).

[89] T. Wang , A. Molassiotis , B. P. M. Chung , and J. Y. Tan , “Unmet Care Needs of Advanced Cancer Patients and Their Informal Caregivers: A Systematic Review,” BMC Palliative Care 17, no. 1 (2018): 96, 10.1186/s12904-018-0346-9. 30037346 PMC6057056

[90] J. A. Su , D. C. Yeh , C. C. Chang , et al., “Depression and Family Support in Breast Cancer Patients,” Neuropsychiatric Disease and Treatment 13 (2017): 2389–2396, 10.2147/ndt.s135624. 28979126 PMC5602463

[91] D. K. Sari , R. Dewi , and W. Daulay , “Association Between Family Support, Coping Strategies and Anxiety in Cancer Patients Undergoing Chemotherapy at General Hospital in Medan, North Sumatera, Indonesia,” Asian Pacific Journal of Cancer Prevention 20, no. 10 (2019): 3015–3019, 10.31557/apjcp.2019.20.10.3015. 31653149 PMC6982683

[92] Mental Health Support for Cancer Patients: Why Emotional Well‐Being Matters,” American Oncology Institute (2024).

[93] W. Zhu , “The Impact of Social Support on the Mental Health of Cancer Patients: Evidence From China,” Psycho‐Oncology 18, no. 1 (2024): 69–77, 10.32604/po.2023.046593.

[94] E. Chong , L. Crowe , K. Mentor , S. Pandanaboyana , and L. Sharp , “Systematic Review of Caregiver Burden, Unmet Needs and Quality‐of‐Life Among Informal Caregivers of Patients With Pancreatic Cancer,” Supportive Care in Cancer 31, no. 1 (2022): 74, 10.1007/s00520-022-07468-7. 36544073 PMC9771849

