# Supplementary material for: Psychosocial Outcomes in Patients Who Participated in a Hospital‐Based Family Involvement Program After Major Abdominal Oncological Surgery: A Preplanned Secondary Analysis of a Prospective Cohort Study
Source: Psychooncology. 2026 Jan 1;35(1):e70373. doi: 10.1002/pon.70373 (PMC12757203; doi:10.1002/pon.70373)
Supplement: Supplementary file 1 — Supporting Information S1 [file PON-35-e70373-s001.docx]

**Supplementary**

**Psychosocial outcomes in patients who participated in a hospital-based family involvement program after major abdominal oncological surgery: a preplanned secondary analysis of a prospective cohort study**

**Supplementary**


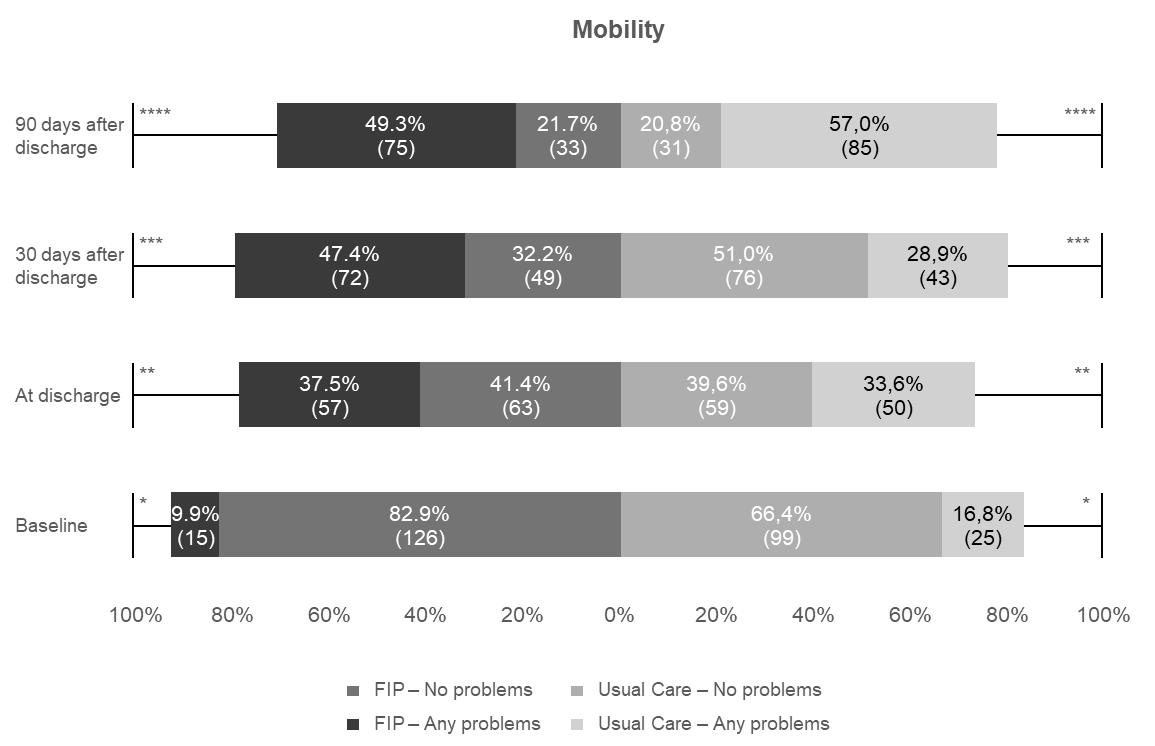


Figure S1A: The mobility dimension scores as one of the five dimension of the health related quality of life questionnaire (EQ5D5L), measured at aimed follow up points during the study. Numbers are presented as percentages, with the exact number of patients presented in parentheses.

*Missing at baseline, N=numbers of patients: FIP N=31, usual care N=30.

**Missing at discharge, N=numbers of patients: FIP N=32, usual care N=40.

***Missing at 30days after discharge, N=numbers of patients: FIP N=31, usual care N=30.

****Missing at 90 days after discharge, N= numbers of patients: FIP N=44, usual care N=33.


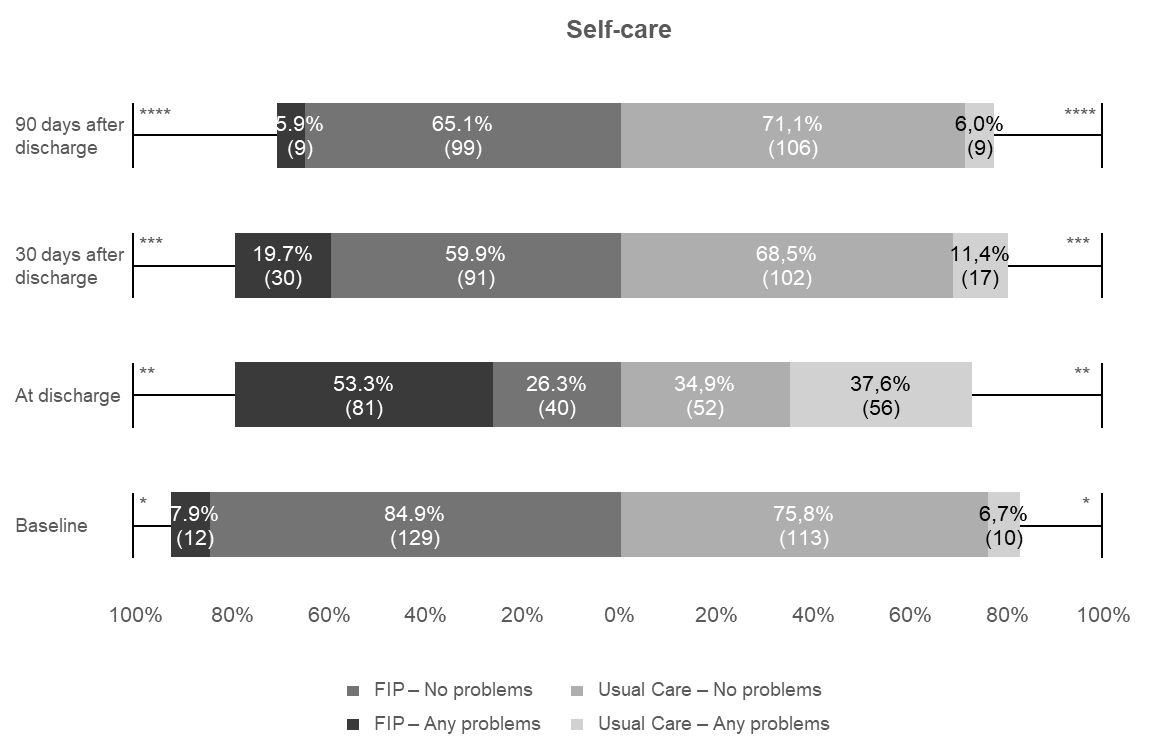


Figure S1B: The self-care dimension scores as one of the five dimension of the health related quality of life questionnaire (EQ5D5L), measured at aimed follow up points during the study. Numbers are presented as percentages, with the exact number of patients presented between parentheses.

*Missing at baseline, N=numbers of patients: FIP N=11, usual care N=26.

**Missing at discharge, N=numbers of patients: FIP N=31, usual care N=41.

***Missing at 30days after discharge, N=numbers of patients: FIP N=31, usual care N=30.

****Missing at 90 days after discharge, N= numbers of patients: FIP N=44, usual care N=34.


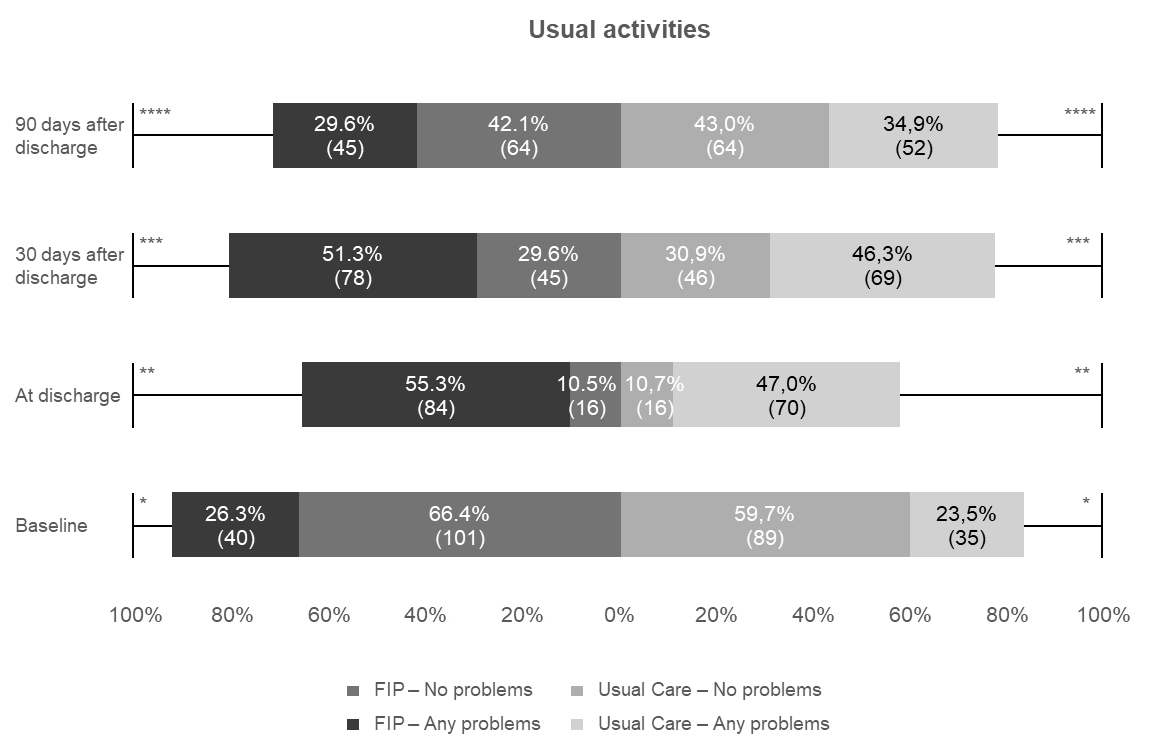


Figure S1C: The usual activities dimension scores as one of the five dimension of the health related quality of life questionnaire (EQ5D5L), measured at aimed follow up points during the study. Numbers are presented as percentages, with the exact number of patients presented in parentheses.

*Missing at baseline, N=numbers of patients: FIP N=11, usual care N=25.

**Missing at discharge, N=numbers of patients: FIP N=52 , usual care N=63.

***Missing at 30days after discharge, N=numbers of patients: FIP N=29, usual care N=34.

****Missing at 90 days after discharge, N= numbers of patients: FIP N=43, usual care N=33.


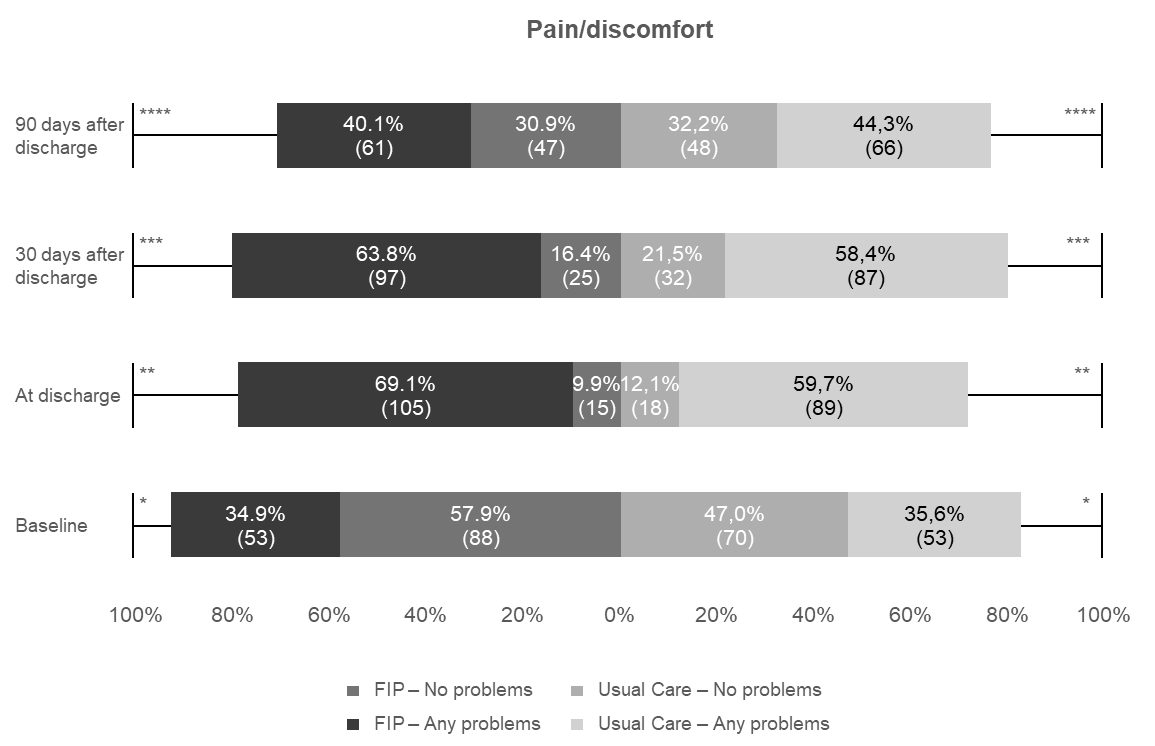


Figure S1D: The pain/discomfort dimension scores as one of the five dimension of the health related quality of life questionnaire (EQ5D5L), measured at aimed follow up points during the study. Numbers are presented as percentages, with the exact number of patients presented in parentheses.

*Missing at baseline, N=numbers of patients: FIP N=11, usual care N=26.

**Missing at discharge, N=numbers of patients: FIP N=32, usual care N=42.

***Missing at 30days after discharge, N=numbers of patients: FIP N=30, usual care N=30.

****Missing at 90 days after discharge, N= numbers of patients: FIP N=44, usual care N=35.


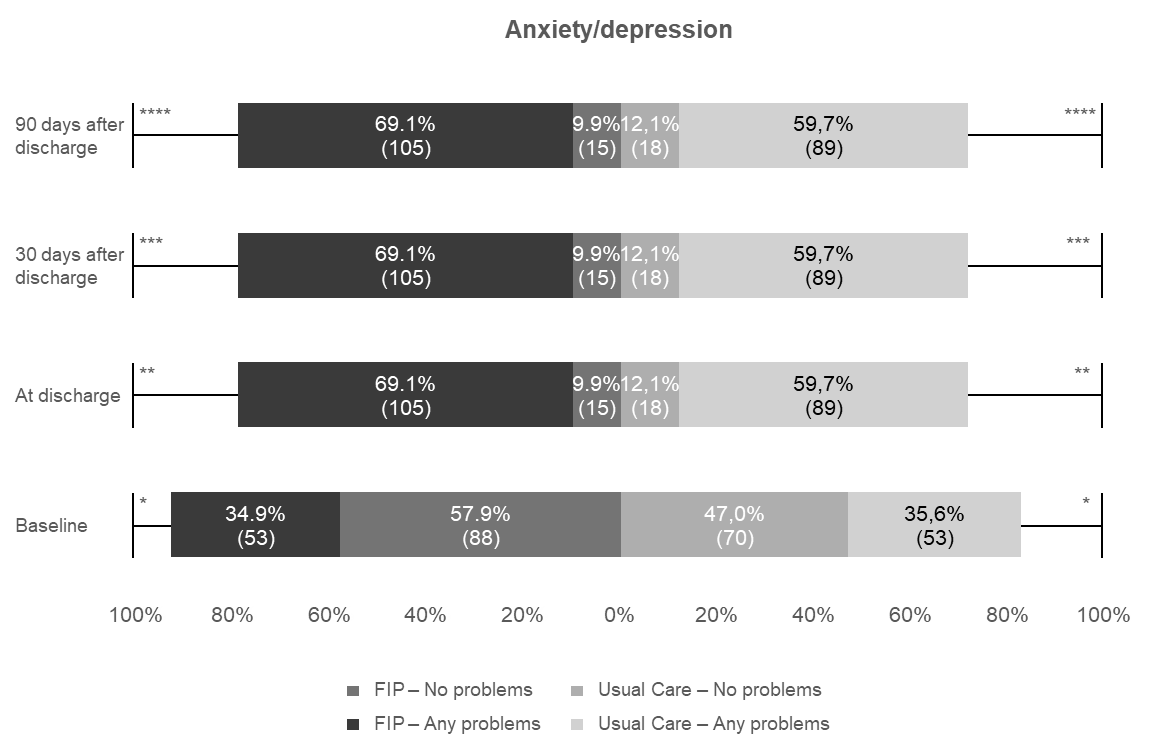


Figure S1E: The anxiety/depression dimension scores as one of the five dimension of the health related quality of life questionnaire (EQ5D5L), measured at aimed follow up points during the study. Numbers are presented as percentages, with the exact number of patients presented in parentheses.

*Missing at baseline, N=numbers of patients: FIP N=11, usual care N=26.

**Missing at discharge, N=numbers of patients: FIP N=32, usual care N=42.

***Missing at 30days after discharge, N=numbers of patients: FIP N=32, usual care N=42.

****Missing at 90 days after discharge, N= numbers of patients: FIP N=32, usual care N=42.
